# Radiotherapy in the Management of Non-Metastatic Inflammatory Breast Cancers: A Retrospective Observational Study

**DOI:** 10.3390/cancers14010107

**Published:** 2021-12-27

**Authors:** Benjamin Nicaise, Pierre Loap, Delphine Loirat, Fatima Laki, Jean-Yves Pierga, Alain Fourquet, Youlia Kirova

**Affiliations:** 1 Department of Radiation Oncology, Institut Curie, 75005 Paris, France; pierre.loap@curie.fr (P.L.); alain.fourquet@curie.fr (A.F.); 2 Faculty of Medicine, Sorbonne Université, 75006 Paris, France; 3 Department of Medical Oncology, Institut Curie, 75005 Paris, France; delphine.loirat@curie.fr (D.L.); jean-yves.pierga@curie.fr (J.-Y.P.); 4 Department of Surgery, Institut Curie, 75005 Paris, France; fatima.laki@curie.fr; 5 Faculty of Medicine, Université de Versailles Saint-Quentin-en-Yvelines, 78000 Versailles, France

**Keywords:** inflammatory breast cancers, triple-negative breast cancers, post-mastectomy radiation therapy

## Abstract

**Simple Summary:**

Inflammatory breast cancer (IBC) is a rare and aggressive clinicopathological presentation of breast cancer. The treatment of non-metastatic IBC usually consists of neoadjuvant systemic therapy, total mastectomy with axillary lymph node dissection, and adjuvant radiotherapy. This single-center retrospective study aims to assess the clinical outcomes of curative-intent multidisciplinary treatment of non-metastatic IBC. We identified 113 patients with a 5-year overall survival of 78.0% and a 5-year locoregional recurrence-free survival of 85.2%, highlighting a high locoregional control with standard fractionation 3D electron or photon therapy in our cohort, despite a pejorative overall survival.

**Abstract:**

(1) Background: Inflammatory breast cancers (IBC) are characterized by a poor prognosis. This retrospective study aims to describe the clinical outcomes of non-metastatic IBC patients treated with a multidisciplinary approach with neo-adjuvant chemotherapy, surgery, and radiotherapy. (2) Methods: This single-center retrospective study included all women patients diagnosed with non-metastatic IBC between January 2010 and January 2018 at the Institut Curie (Paris, France) and treated with neoadjuvant chemotherapy, surgery, and radiotherapy. Overall survival (OS), disease-free survival (DFS), and locoregional free survival (LRRFS) were calculated from the time of diagnosis. Prognostic factors for patient survival were analyzed based on univariate and multivariate regressions. (3) Results: We identified 113 patients with a median age of 51 years. 79.7% had node-positive tumors; triple-negative breast cancers (TNBC) represented 34.6% of the cases. A large majority of patients (91.2%) received adjuvant post-mastectomy while ten patients (8.8%) received preoperative radiotherapy. Non-pathological complete response (non-pCR) was observed in 67.3% of patients. Radiotherapy delivered a median dose of 50 Gy to the breast or the chest wall in 25 fractions. With a median follow-up of 54 months, 5-year OS, DFS and LRRFS were 78% (CI: 70.1–86.8%), 68.1% (59.6–77.7%), and 85.2% (78.4–92.7%), respectively. In multivariate analysis, non-pCR was an adverse prognosis factor for OS, DFS, and LRRFS; pre-operative radiotherapy was an adverse prognosis factor for OS and DFS. Radiation-related adverse events were limited to acute skin toxicity (22% of Grade 2 and 2% of grade 3 dermatitis); no late radiation-induced toxicity was reported. (4) Conclusions: High locoregional control could be achieved with multidisciplinary management of non-metastatic IBC, suggesting the anti-tumor efficacy of radiotherapy in this rare but pejorative clinicopathological presentation. While comparing favorably with historical cohorts, OS and DFS could be potentially improved in the future with the use of new systemic treatments, such as PARP-inhibitors or immunotherapy.

## 1. Introduction

Inflammatory breast cancer (IBC) is a rare clinicopathological presentation of breast cancers (BC), representing only 2.0% of all incident BC cases [1], characteristic pathological features of which include diffuse tumor emboli in dermal lymphatic vessels. This entity is classified as T4d in the AJCC 8th edition TNM [2]: clinical features must include erythema, edema, or *peau d’orange* encompassing more than one-third of the breast and must have developed in the last six months, thus excluding secondary inflammation related to locally advanced breast cancers.

IBC are characterized by a poor prognosis compared to non-inflammatory BC, with 5-year overall survivals ranging from 50 to 65% in historical cohorts [3,4,5,6,7,8,9], because of a high risk of rapid progression and distant dissemination. Yet almost 70% of patients are diagnosed at a locoregional stage [10] and thus are candidates for a curative intent treatment. Treatment of non-metastatic IBC usually includes neo-adjuvant chemotherapy, followed by total mastectomy with axillary lymph node dissection and adjuvant radiotherapy. This multidisciplinary management often called trimodality is the standard of care, which is supported by retrospective studies showing a survival benefit with this strategy [5,11].

Neo-adjuvant systemic treatment regimens are the same as recommended in non-IBC.

Radiotherapy of the chest wall and lymph node areas is usually performed via standard fractionation [4,6]. A technique using electron field plus bolus for chest wall irradiation and a mix of photons and electrons without bolus for the internal mammary chain (IMC) has been used at our institution since 2007 [12]. This technique showed a benefit on locoregional control at 5 years compared to chest wall irradiation with photons [13], but T4 accounted for only 1% of our retrospective cohort.

Here we present a retrospective observational study aiming to describe the clinical outcomes of non-metastatic IBC patients treated with a multidisciplinary approach at our institution in the past decade.

## 2. Materials and Method

We identified women patients over the age of 18 who were diagnosed between January 2010 and January 2018 and were treated at our institution with multidisciplinary management for T4d non-metastatic breast cancer according to the AJCC 8th edition TNM. We excluded patients with metastatic disease at diagnosis or before the beginning of locoregional treatment, and patients who did not undergo either neo-adjuvant systemic therapy or radiotherapy.

Institutional review board approval was obtained (CRI-DATA#200146).

Receptor status was based on the biopsy made at our center. If a patient was first diagnosed outside without a second biopsy or proofreading at our center, we relied on the results found by private practice pathologists. Hormone receptor expression was considered positive if estrogen receptor (ER) or progesterone receptor (PR) expression was 1% or more. Tumors were considered HER2 positive if the immunohistochemical staining score was 3+ or if FISH was positive.

Nodal status was defined according to clinical data and the combination of the different imaging modalities available (mammogram, echogram, MRI, and/or PET-CT). If a mass was visible on breast imaging, its largest diameter was recorded (in millimeter) as well as the response to neoadjuvant systemic treatment according to RECIST.

Precise treatment modalities were reported: type of chemotherapy regimen in neo-adjuvant, concomitant to radiotherapy or adjuvant setting; use of HER2 inhibitors and hormone therapy; type of surgery; radiotherapy with prescribed dose, fractionation, target volumes, use of electron or photon fields and technique.

The dosimetric technique used at one of our sites was 3D-conformal electron radiotherapy to the chest wall with bolus adapted to chest wall thickness. Bolus was cut in its medial part to allow electrons to reach IMC. A photon field was added to target IMC while the other lymph node areas were treated exclusively with photons. Patients treated at another of our sites received chest wall irradiation with tangential photon field without bolus and a contribution of photons and electrons to IMC. Rotational IMRT techniques were used in case of particular anatomy or large volumes potentially leading to dosimetric problems. Irradiation of Berg level I was performed in case of positivity of more than 50% of lymph nodes in axillary dissection.

Some patients underwent standard photon radiotherapy outside of our institution.

Pathological response on the surgical piece was defined preferentially by RCB score, with an RCB score of 0 corresponding to pathological complete response (pCR). Whenever RCB score was not available or computable, pathological complete response was defined according to Sataloff classification and must therefore correspond to TA (total or near therapeutic effect on primary tumor) and NA (evidence of therapeutic effect without residual tumor on axillary nodes).

Survival endpoints were calculated from the time of diagnosis. We used definitions of the STEEP statement [14]. Overall survival events were: death from breast cancer and death from non-breast cancer cause. Disease-free survival (DFS) events were: invasive (or in situ) ipsilateral breast tumor recurrence, locoregional invasive recurrence, distant recurrence, death from breast cancer, death from non-breast cancer cause, or second primary invasive non-breast cancer. Locoregional recurrence-free survival (LRRFS) events were: invasive (or in situ) ipsilateral breast tumor recurrence and locoregional invasive recurrence.

Acute and late radiotherapy-related toxicity were retrospectively recorded according to Common terminology criteria for adverse events version 5 (CTCAE v5).

For the description of population, treatments, and toxicity, quantitative parameters were described with median value and range (minimum–maximum) while qualitative parameters were mentioned with proportions. Survival analyses were performed using Kaplan–Meyer method. Relationship between LRRFS, DFS, OS, and clinical characteristics were evaluated using the Cox regression model: we performed multivariate analysis adjusted for age, clinical nodal status, tumoral diameter on imaging (mm), histologic subtype, histologic grade, RECIST response, pathological response, a capsular rupture in lymph nodes, radiotherapy setting, type of radiotherapy beam (electron of photon) and interruption of radiotherapy. The statistical significance threshold was set at 0.05. Statistical analyses were performed using R 4.0.3 software, based on the “epiR” and “survminer” packages.

## 3. Results

### 3.1. Population

From January 2010 to January 2018, 113 patients were treated for a non-metastatic IBC in a curative intent with neo-adjuvant systemic treatment, radiotherapy with or without surgery (Table 1).

The median age was 51 years (range 26–89 years). Median Body mass index (BMI) was located in overweight (26.7 (18.4–45.0)). Five patients were carriers of germline BRCA1 mutations (4.4%), while one patient had a germline BRCA2 mutation identified (0.1%).

Ninety patients (79.7%) had a regional nodal involvement at diagnosis. The large majority was ductal invasive carcinoma (93.8%). Ki67 proliferation index was high (median 40%) and there was a majority of grade III tumors (60.2%).

The histologic subtype repartition was as follows: 44.2% hormone receptor-positive/HER negative (HR+/HER2−), 34.6% triple-negative (TNBC), and 21.2% HER2 positive (HER2+).

### 3.2. Treatments

All but 3 patients underwent a total mastectomy and all but 4 underwent axillary dissection (Table 2).

According to eligibility criteria, neo-adjuvant systemic treatment was administered in all patients, with a high proportion of taxanes and anthracycline-containing regimens. Chemotherapy was administered concurrently with radiotherapy in 17 patients (15.0%) predominantly with 5FU-vinorelbine (12 patients) or capecitabine alone (5 patients). Adjuvant chemotherapy was given in 6 patients including 3 who received adjuvant capecitabine. Fifty-three percent received adjuvant hormone therapy.

Twenty-four patients received HER2 inhibitors. Six patients received an association of trastuzumab and pertuzumab in the neoadjuvant setting. All of these patients had maintenance with trastuzumab alone except one who received TDM-1.

According to eligibility criteria, radiotherapy was given to all patients but only ten patients (8.8%) underwent preoperative radiotherapy. Radiotherapy delivered a median dose of 50 Gy (36–52) with a median dose per fraction of 2 Gy (18–29). Berg lymph node levels II, III, and IV and internal mammary chain were targeted in the majority of patients, and level I was targeted in only 21.2%. Very few boosts to the scar or nodules were given (5.3%). The main technique was 3D-conformal radiotherapy (90.3%). Thirty-four patients received an electron field to the chest wall (with photons and electrons to the IMC and photons to the other node areas) according to the dosimetric technique described earlier. The other 65 patients with dosimetric data available received photon fields without electrons to the chest wall (with photons and electrons to the IMC in 30 patients, electrons alone to the IMC in 4 patients, photons alone to the IMC in 24 patients, and photons to the other node areas). Eleven patients were treated with rotational intensity-modulated radiation therapy (VMAT or helical tomotherapy).

### 3.3. Outcomes

**Figure 1:**
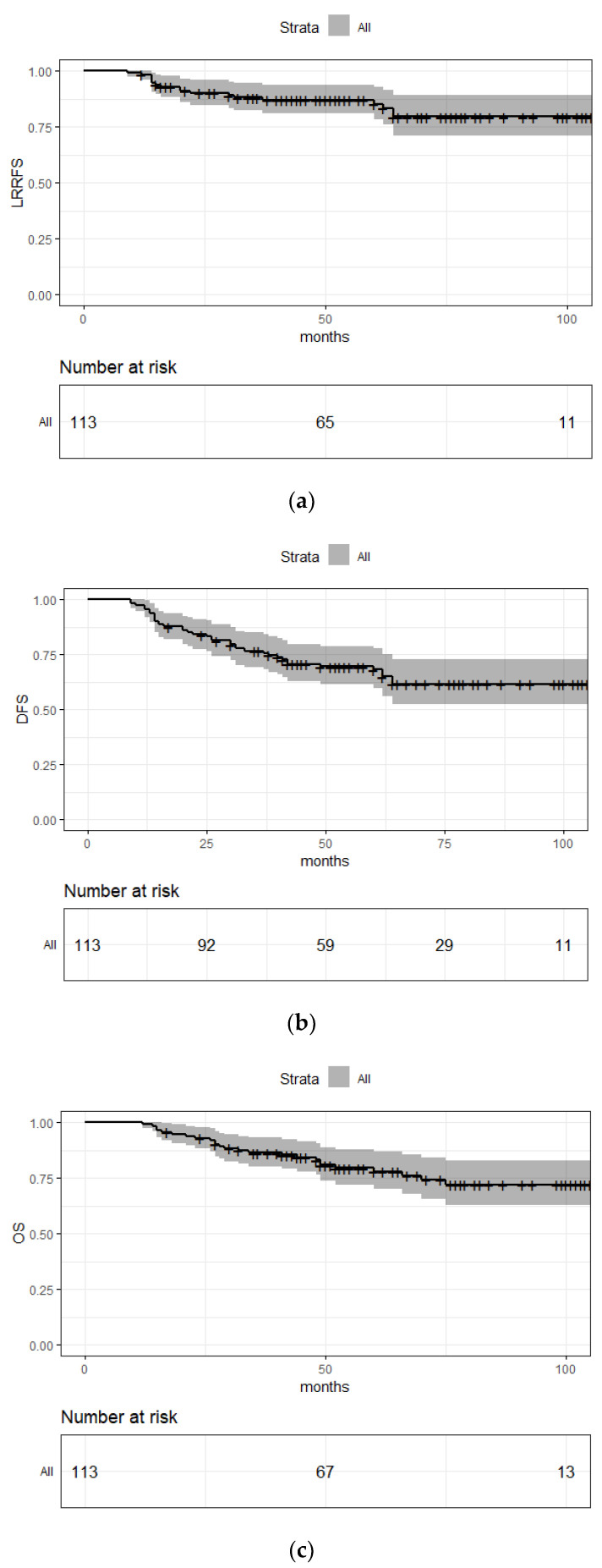
Survival outcomes: (**a**) LRRFS; (**b**) DFS; (**c**) OS.

With a median follow-up of 54 months, 5-year OS, DFS, and LRRFS were 78% (CI: 70.1–86.8%), 68.1% (59.6–77.7%), and 85.2% (78.4–92.7%), respectively (Figure 1). A pathological complete response rate of 32.7% was observed.

Results from the univariate analysis are reported in Table 3; variables that were significant in univariate analysis were included in multivariate analysis.

Non-pCR was an adverse prognosis factor for OS, DFS and LRRFS (*p* < 0.01, *p* < 0.01 and *p* < 0.01, respectively) both in univariate and multivariate analyses (multivariate adjusted HR = 4.40 for OS (95%CI: 1.42–21.85; *p* = 0.007); HR = 6.06 for DFS (95%CI: 2.31–22.39; *p* = 0.001); HR = 17.34 for LRRFS (95%CI: 2.29–2222.44; *p* = 0.002)). Pre-operative radiotherapy was an adverse prognosis factor for OS and DFS (*p* < 0.01 and *p* < 0.01, respectively), both in univariate and multivariate analyses (multivariate adjusted HR = 5.22 for OS (95%CI: 1.94–12.38; *p* = 0.02); HR = 3.81 for DFS (95%CI: 1.58–8.07; *p* = 0.002). Suspension of radiotherapy was an adverse prognosis factor for OS in univariate analysis but did not remain significant in multivariate analysis (multivariate adjusted HR = 2.66 (95%CI: 0.93–6.43; *p* = 0.07)); capsular rupture in lymph node metastases was an adverse prognosis factor for LRRFS in univariate analysis but did not remain significant in multivariate analysis (multivariate adjusted HR = 2.12 (95%CI: 0.77–5.32; *p* = 0.14)). Neither diameter of the mass on breast imaging or use of electron beams for chest wall radiotherapy were found to be predictive of survival (Table 3).

**Figure A1:**
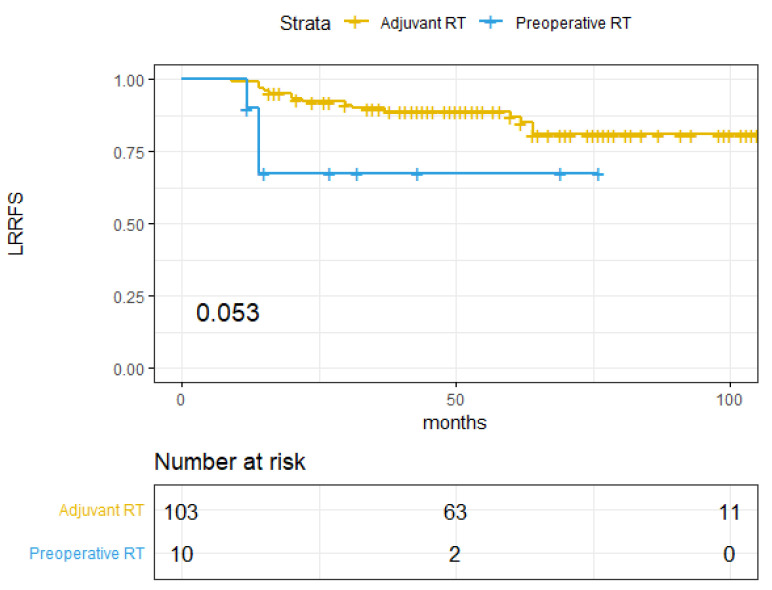
LRRFS with adjuvant radiotherapy (5-y LRRFS: 87.0% [80.2–94.4%]) vs. with preoperative radiotherapy (67.5% [43–100%]).

**Figure A2:**
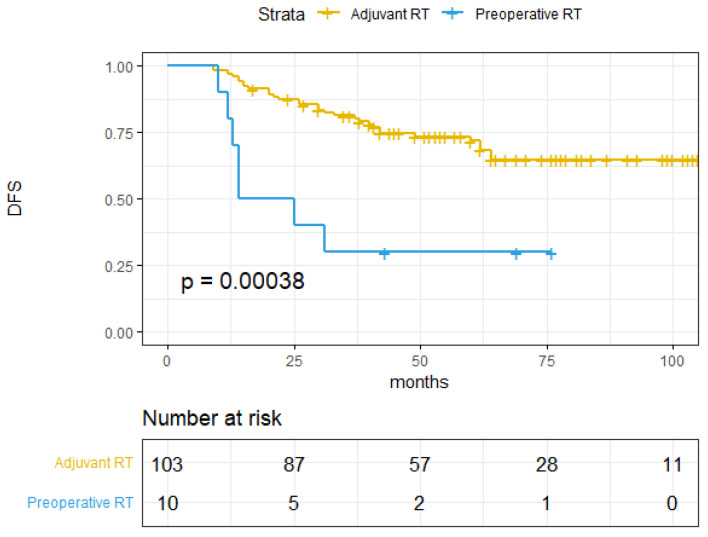
DFS with adjuvant radiotherapy (5-y DFS: 71.8% [63.1–81.6%]) vs. with preoperative radiotherapy (30.0% [11.6–77.3%]).

**Figure A3:**
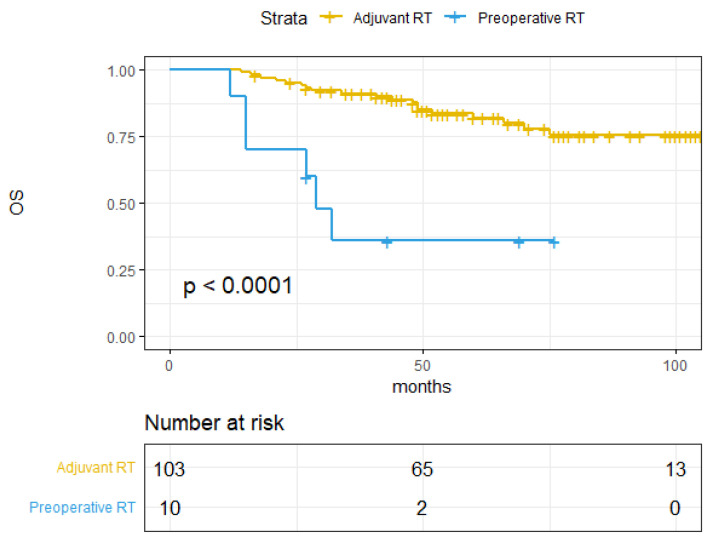
OS with adjuvant radiotherapy (5-y OS: 81.9% [74.1–90.6%]) vs. with preoperative radiotherapy (36.0% [15.0–86.5%]).

**Figure A4:**
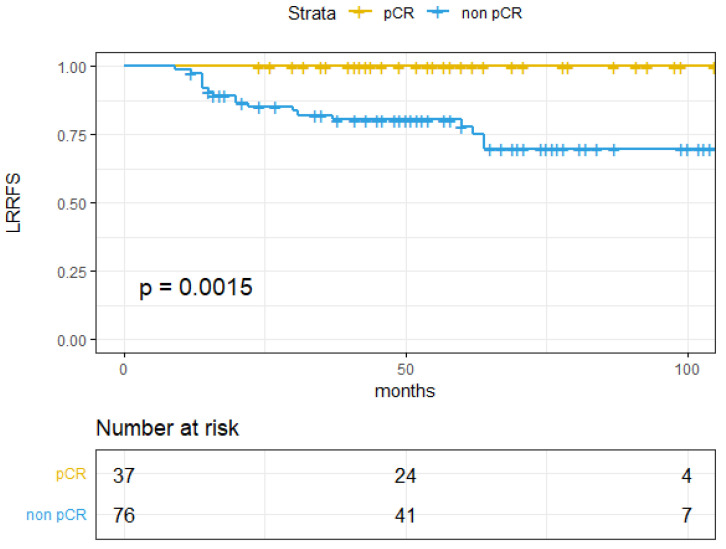
LRRFS in patients achieving pCR (5-y LRRFS: 100% [100–100%]) and those not achieving pCR (77.9% [68.3–88.8%]).

**Figure A5:**
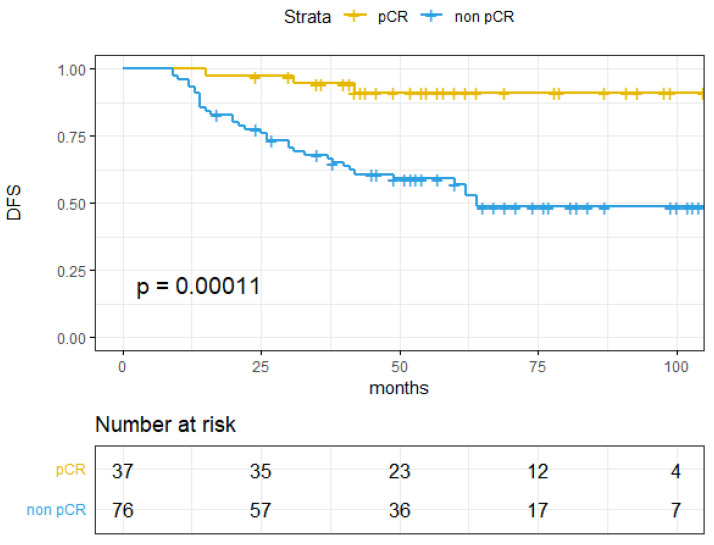
DFS in patients achieving pCR (5-y DFS: 91.2% [82.1–100%]) and those not achieving pCR (57.1% [46.6–70.0%]).

**Figure A6:**
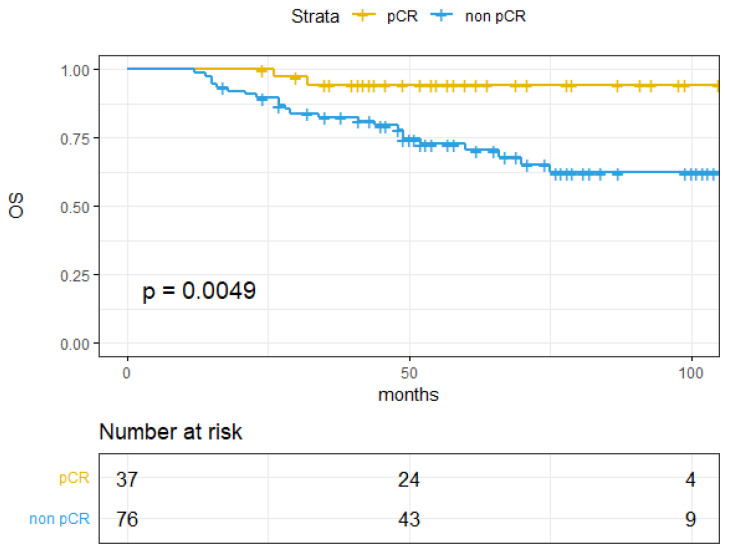
OS in patients achieving pCR (5-y OS: 94.4% [87.1–100%]) and those not achieving pCR (70.5% [59.9–82.3%]).

Survival curves in adjuvant vs. preoperative radiotherapy setting (Figure A1, Figure A2 and Figure A3) and in pCR vs. non-pCR subgroup (Figure A4, Figure A5 and Figure A6) are shown in Appendix A.

### 3.4. Toxicity

Radiation-related adverse events were limited to acute skin toxicity (22% of grade 2 and 2% of grade 3 dermatitis); no late cardiovascular or pulmonary radiation-induced toxicity was reported (Table 4). Because of radiodermatitis or pain, ten patients had a temporary interruption of radiotherapy or a cancellation of one or two fractions.

## 4. Discussion

Our population was quite similar to that of previous cohort studies.

The majority of patients were diagnosed with aggressive clinicopathological features (high prevalence of clinical nodal involvement and grade III).

Consistently with what Gutierrez Barrera et al. reported [15], germline BRCA mutations are not more prevalent in IBC than in the non-IBC population. Lobular invasive carcinomas account for about 6% of our cohort, which is lower than the 10–15% reported in non-IBC [16,17].

It is important to notice that, as reported elsewhere, TNBC and HER2+ subtypes are more prevalent in IBC than in non-IBC [8,9,18,19]. We even had a tendency towards an overrepresentation of TNBC (34.6%) compared to previous cohorts which reported rates of TNBC not exceeding 26%. This might be explained by our relatively small sample, a tertiary referral hospital bias, and maybe a different ethnic distribution (Biswas et al. reported the highest frequency of TNBC among Black patients [8]).

Patients in our cohort benefited from progress made in the systemic treatment of breast cancers with a high proportion of taxane and anthracycline-containing regimens. HER2 inhibitors were used when indicated. Very few patients not achieving pCR received adjuvant capecitabine, given that the results of the CREATE-X study were published recently in 2017 [20]. Similarly, only one patient with HER2+ IBC not in pCR after neoadjuvant systemic treatment underwent adjuvant TDM-1, based on the KATHERINE trial published in 2019 [21].

One-third of our patients received electron-based irradiation to the chest wall while the other received photons to the chest wall, using standard fractionation.

Five-year OS, DFS and LRRFS were 78% (CI: 70.1–86.8%), 68.1% (59.6–77.7%), and 85.2% (78.4–92.7%), respectively. These survival outcomes are much better than in historical cohorts, which reported 5-year OS between 50 and 65% [3,4,5,6,8,9].

These good results might be explained by the progress made in systemic treatment. Specific treatments for IBC have been tried, such as the addition of bevacizumab (BEVERLY-1 and BEVERLY-2 trials [22,23]) or repeated hematopoietic stem cell support (PEGASE 02, 05, and 07 [24,25]), but both failed to become standards of care because of the absence or paucity of clinical benefit with increased toxicity. Nevertheless, non-metastatic IBC management benefited from progress made in non-IBC, particularly from HER2 inhibitors.

Our good survival results might also be explained by the systematic use of multidisciplinary management (trimodality), while in the cohort of non-metastatic IBC reported by van Uden et al. [9], trimodality was used in only 53% of patients. Another example is the cohort of patients treated with radiotherapy between 1977 and 2004 at MD Anderson Cancer Center, presented by Bristol [3], which showed a 5-year OS of 51%. Twenty-five percent of patients did not receive complete trimodality treatment in this study because of disease progression during treatment and very few received HER2-targeted therapy.

Although our survival outcomes were better than previously reported in the literature of IBC, it remains poorer than in non-IBC. Comparatively, Grellier et al. found a 5-year OS of 90.9% and a 5-year LRRFS of 90.0% in women treated with post-mastectomy electron radiotherapy for non-IBC [12].

Our high locoregional control is similar to that reported in previous radiotherapy studies in which 5-year LRRFS ranged from 79 to 87% [3,4,5,6], suggesting the validity of radiotherapy techniques used at our institution.

Boulle et al. reported a reduced risk of locoregional recurrence with electrons compared to photons for post-mastectomy irradiation of non-IBC at our center [13]. Here we reported no statistically significant difference between these two irradiation modalities in IBC. This might be explained by the clinical relevance of lymph node metastatic involvement in IBC, which is targeted by photons in both irradiation modalities.

Bristol et al. did not find a benefit of accelerated hyperfractionated radiotherapy but found a better locoregional control with a dose escalation to 66 Gy compared to 60 Gy in women under the age of 45 or with positive margins or not having responded to chemotherapy [3]. In our cohort, patients were treated with standard fractionation (median dose 50 Gy in 2 Gy fractions) and we found a similar locoregional control rate compared to the 84% 5-year LRRFS reported at MD Anderson. Young age was not found to be an adverse prognosis factor in univariate or multivariate analysis in our cohort.

The diameter of the mass on breast imaging was not found to be a prognosis factor. This highlights the relevance of TNM classification: T4d breast cancers should not be subdivided into different classes according to the diameter of the tumor.

The pathological complete response rate was 32.7% in our cohort, similar to the 31% reported in both IBC and locally advanced breast cancers by Monneur et al. [26]. Pathological complete response was found to be associated with OS, DFS, and LRRFS. This has been previously observed in IBC studies [8,9], consistently with what is already known in non-IBC [27].

Preoperative radiotherapy was found to be associated with a poorer DFS and OS in multivariate analysis. This can be explained by possible confounding factors not taken into account in regression analysis because preoperative radiotherapy was usually performed in our center for patients with pejorative prognosis who poorly responded to chemotherapy.

The toxicity profile of radiotherapy was good in our cohort. In particular, no late cardiovascular or pulmonary radiation-induced toxicity was reported. Yet the follow-up (54 months) might be a bit short to detect cardiovascular events in our cohort of 113 patients.

Suspension of radiotherapy because of radiodermatitis was found to be associated with poorer OS in univariate analysis and it was close to statistical significance in multivariate analysis, suggesting that radiotherapy should not be interrupted because of manageable acute skin toxicity.

## 5. Conclusions

Despite the limitation due to the retrospective nature of this study, we reported high locoregional control with multidisciplinary management of non-metastatic inflammatory breast cancers and the use of either photon or electron radiotherapy. OS and DFS were found to be better than in historical cohorts, probably because of progress made in systemic treatment including HER2 targeted therapy. Yet, survival remains poorer than in non-inflammatory breast cancers, particularly in the non-pCR group, and could be improved in the future with PARP inhibitors (RADIOPARP trial [28]) or immune therapy (PELICAN-IPC 2015-016/Oncodistinct-003 trial [29]).

## Figures and Tables

**Table 1:** Demographic characteristics.

	Median (Range)	Number of Patients (n = 113), Proportion	
**Age (median (range))**	51 (26–89)		
**BMI (median (range))**	26.7 (18.4–45.0)		
**Genetic predisposition**			
BRCA1 mutation		5	4.4%
BRCA2 mutation		1	0.1%
Unknown		107	96.5%
**Clinical stage**			
T4d N−		23	20.3%
T4d N+		90	79.7%
**Histological type**			
Ductal		106	93.8%
Lobular		7	6.2%
**Ki67 (median (range))**	40 (0–95)		
**Histological grade (SBR)**			
I		3	2.7%
II		40	35.4%
III		68	60.2%
NA ^1^		2	1.7%
**Receptor status**			
HR+/HER2−		50	44.2%
HER2+		24	21.2%
HR+/HER2+		12	10.6%
HR−/HER2+		12	10.6%
Triple-negative		39	34.6%
**Pathological response**			
Pathological complete response (pCR)		37	32.7%
Non-pCR		76	67.3%

^1^ data not available.

**Table 2:** Treatment details.

	Median (Range)	Number of Patients (n = 113), Proportion	
**Systemic treatment**			
**Neoadjuvant**		113	100%
Anthracycline-containing		104	92.0%
Taxane-containing		111	98.2%
Cyclophosphamide-containing		108	95.6%
5FU-containing		68	60.2%
Carboplatin-containing		1	0.9%
Bevacizumab-containing		2	1.8%
**Concomitant with radiotherapy**		17	15.0%
5FU-Vinorelbine		12	
Capecitabine		5	
Bevacizumab		1	
**Adjuvant**		6	5.3%
Capecitabine		3	2,7%
Other ^1^		3	2.7%
**HER2 inhibitors**		24	21.2%
Trastuzumab-Pertuzumab		6	5.3%
TDM1		1	0.9%
**Hormone therapy**		62	5.3%
**Surgery**			
Mastectomy		110	97.3%
Breast-conserving surgery		2	2.7%
Axillary dissection		109	96.5%
Sentinel lymph node biopsy		3	3.5%
No surgery		1	0.1%
**Radiotherapy**			
**Setting**			
Preoperative		10	8.8%
Adjuvant		103	91.2%
**Regimen**			
Dose (Gy (range))	50 (36–52)		
Fractions (range)	25 (18–29)		
**Target volumes**			
Chest wall/breast		113	100%
Boost (scar/nodules)		6 (4/2)	5.3%
Berg’s I lymph node		24	21.2%
Berg’s II-III lymph node		100	88.5%
Berg’s IV lymph node		101	89.4%
Internal mammary chain		96	85.0%
**Technique**			
3D		102	90.3%
Electrons (chest wall)		34	30.1%
Photons (chest wall)		52	46%
With photons and electrons to IMC		30	26.5%
With electrons to IMC		4	3.5%
With photons to IMC		13	11.5%
NA ^2^		14	12.4%
VMAT		3	2.7%
Tomotherapy		8	7.0%

^1^ Olaparib (1), everolimus (1), oral cyclophosphamide (1); ^2^ complete dosimetric data not available.

**Table 3:** Univariate analyses of prognostic factors for locoregional recurrences, disease-free survival, and overall survival.

	5-Year LRRFS			5-Year DFS			5-Year OS		
	HR		*p*-Value	HR		*p*-Value	HR		*p* Value
Age	1.09 (0.43–2.74)		0.859	1.14 (0.6–2.15)		0.687	0.85 (0.39–1.88)		0.69
<50 years		85.77% (75.91–96.91%)			69.36% (57.87–83.13%)			73.74% (62.44–87.09%)	
>50 years		84.25% (74.77–94.93%)			65.95% (53.89-80.72%)			82.74% (72.31–94.66%)	
cN	2.17 (0.5–9.45)		0.301	0.79 (0.37–1.66)		0.526	0.45 (0.2–1.01)		0.052
N-		91.3% (80.49–100%)			57.54% (39.55–83.72%)			60.47% (42.14–86.78%)	
N+		83.71% (75.7–92.57%)			70.63% (61.41–81.24%)			82.87% (74.97–91.59%)	
Tumoral diameter on imaging (mm)	1 (0.98–1.02)		0.910	0.99 (0.98–1.01)		0.367	1 (0.98–1.02)		0.763
Grade	1.15 (0.43–3.08)		0.778	0.75 (0.39–1.43)		0.380	0.82 (0.36–1.84)		0.623
I-II		83.88% (70.32–100%)			59.4% (44.5–79.29%)			78.65% (66.22–93.4%)	
III		84.5% (76.07–93.86%)			71.45% (61.35–83.22%)			77.14% (67.11–88.67%)	
Histology	1.72 (0.68–4.35)		0.255	1.46 (0.76–2.81)		0.253	2.12 (0.97–4.66)		0.061
HR+ or HER2+		88.99% (81.3–97.41%)			71.53% (61.27–83.5%)			83.64% (74.65–93.72%)	
TNBC		77.93% (65.46–92.77%)			61.54% (48.02–78.87%)			67.76% (54.06–84.92%)	
RECIST	2.04 (0.52–7.97)		0.304	1.79 (0.74–4.31)		0.193	2.26 (0.77–6.64)		0.138
CR/PR		88.82% (80.44–98.06%)			71.86% (61.14–84.45%)			85.19% (76.65–94.68%)	
SD/PD		88.24% (74.18–100%)			63.09% (43.32–91.88%)			72.4% (52.11–100%)	
Tumor response	3.03 (1.47–20.63)		<0.01	7.28 (2.24–23.66)		0.001	6.14 (1.45–26.03)		0.014
pCR		100% (100–100%)			91.18% (82.09–100%)			94.36% (87.07–100%)	
Non-pCR		77.86% (68.26–88.8%)			57.11% (46.6–70%)			70.32% (59.94–82.49%)	
Capsular rupture	3.46 (1.3–9.22)		0.013	1.95 (0.89–4.25)		0.095	1.29 (0.44–3.75)		0.645
No		89.59% (83.67–95.92%)			71.56% (62.97–81.32%)			79.77% (71.62–88.86%)	
Yes		60.11% (37.82–95.55%)			46.75% (26.17–83.53%)			67.53% (45.73–99.74%)	
RT setting	3.42 (0.98–11.89)		0.053	4.03 (1.77–9.2)		0.001	6.03 (2.37–15.32)		<0.01
Adjuvant RT		86.98% (80.16–94.38%)			71.78% (63.14–81.59%)			81.91% (74.08–90.57%)	
Pre-operative RT		67.5% (43.03–100%)			30% (11.64–77.32%)			36% (14.98–86.49%)	
RT interruption	1.39 (0.32–6.05)		0.66	1.72 (0.67–4.41)		0.26	2.81 (1.05–7.48)		0.039
No		86.03% (79.02–93.66%)			69.84% (61.07–79.87%)			81.31% (73.47–89.99%)	
Yes		76.19% (52.08–100%)			50% (26.9–92.93%)			50% (26.9–92.93%)	
RT beam type	0.66 (0.21–2.11)		0.482	0.56 (0.25–1.26)		0.164	0.4 (0.14–1.2)		0.104
Photons		85.58% (76.38–95.89%)			66.87% (55.72–80.25%)			73.66% (62.76–86.46%)	
Electrons		90.8% (81.39–100%)			78.98% (66.25–94.14%)			90.63% (81.03–100%)	

**Table 4:** Toxicity profile.

	Toxicity Grade (CTCAE v5)			
	1	2	3	4–5
**Acute**				
Dermatitis	n = 40 (35%)	n = 25 (22%)	n = 2 (2%)	-
Dysphagia	n = 1 (1%)	-	-	-
Pain	-	n = 1 (1%)	-	-
Cardiovascular	-	-	n = 1 (1%) *	-
**Late**				
Edema	n = 1 (1%)	-	-	-
Pulmonary	n = 1 (1%) **	-	-	-
Asthenia	-	n = 1 (1%)	-	-

* Hypokinetic cardiomyopathy with LADCA (left anterior descending coronary artery) stenosis, not related to RT. ** Bronchial hyperreactivity, not related to RT.

## Data Availability

Data are stored in an institutional database.
